# Persistence of a Core Microbiome Through the Ontogeny of a Multi-Host Parasite

**DOI:** 10.3389/fmicb.2020.00954

**Published:** 2020-05-19

**Authors:** Fátima Jorge, Nolwenn M. Dheilly, Robert Poulin

**Affiliations:** ^1^Department of Zoology, University of Otago, Dunedin, New Zealand; ^2^School of Marine and Atmospheric Sciences, Stony Brook University, Stony Brook, NY, United States; ^3^Unité Génétique Virale de Biosécurité, Laboratoire de Ploufragan-Plouzané, Agence Nationale de Sécurité Sanitaire de l’Alimentation, de l’Environnement et du Travail, Ploufragan, France

**Keywords:** bacterial communities, *Coitocaecum parvum*, holobiont, trematode, vertical transmission

## Abstract

Animal microbiomes influence their development, behavior and interactions with other organisms. Parasitic metazoans also harbor microbial communities; although they are likely to modulate host–parasite interactions, little is known about parasite microbiomes. The persistence of microbial communities throughout the life of a parasite is particularly challenging for helminths with complex life cycles. These parasites undergo major morphological changes during their life, and parasitize host species that are immunologically, physiologically, and phylogenetically very different. Here, using 16S amplicon sequencing, we characterize the microbiome of the trematode *Coitocaecum parvum* across four of its life stages: sporocysts, metacercariae and adults inhabiting (respectively) snails, crustaceans and fish, as well as free-living cercariae. Our results demonstrate that, at each life stage, the parasite possesses a phylogenetically diverse microbiome, distinct from that of its hosts or the external environment. The parasite’s microbiome comprises bacterial taxa specific to each life stage in different hosts, as well as a small core set of taxa that persists across the parasite’s whole life. The apparent existence of an ontogenetically and vertically transmitted core microbiome is supported by the findings that the diversity and taxonomic composition of the microbiome does not vary significantly among life stages, and that the main source of microbial taxa at any life stage is the previous life stage. Our results suggest that microbes are an integrated component of the trematode, possibly shaping its phenotype and host–parasite interactions.

## Introduction

The realization that metazoans harbor rich communities of bacteria and other microbes in their tissues and cells is reshaping our view of what an individual organism actually is, and opening a more holistic perspective on organismal ecology and evolution (McFall-Ngai et al., 2013; Bordenstein and Theis, 2015). There is now overwhelming evidence that microbiomes, i.e., microbial communities associated with animals, play important roles in an animal’s development, health, behavior and interactions with other organisms (Diaz Heijtz et al., 2011; Feldhaar, 2011; Ezenwa et al., 2012). For instance, certain microbes, whether or not they have established a symbiotic relationship with an animal, can affect the expression of its immunity against parasites, and the outcome of host-parasite interactions (Hooper et al., 2012; Koch and Schmid-Hempel, 2012). Parasites too harbor their own microbiomes, with likely implications for their infection success and virulence (Dheilly, 2014; Dheilly et al., 2015a). Although little is known about parasite microbiomes at present (Dheilly et al., 2017), there is evidence that parasitic protozoans carry viruses that modulate their pathogenicity (Ives et al., 2011), and that both arthropod parasites (Wilkinson et al., 2016; Ben-Yosef et al., 2017) and gastrointestinal nematodes (Sinnathamby et al., 2018) harbor at least some symbiotic bacteria transmitted vertically across generations. The obligate nature of these microbe-parasite associations not only opens new avenues for the development of novel chemotherapeutic approaches against parasitic diseases (Castiglioni et al., 2017; Jenkins et al., 2019), but also adds a layer of complexity to host-parasite coevolution and ecology (Dheilly et al., 2019).

Specific symbiotic bacteria have been identified in some multi-host parasites, e.g., *Wolbachia* in filarial nematodes (Bouchery et al., 2013) and *Neorickettsia* in some trematodes (Vaughan et al., 2012). However, helminth parasites with complex life cycles are yet to have their full microbiome characterized at all stages of their life. Although microbial communities persist for most of the lifetime in some organisms (Faith et al., 2013) but not others (e.g., Kolodny et al., 2019; Vijayan et al., 2019), multi-host helminths present particular challenges for the temporal maintenance of microbiomes. Throughout their ontogeny, these helminths undergo major morphological changes, and drastic changes in living conditions as they transfer from one host species to a completely different one, with each host species possessing its own set of immunological defenses. Their mode of reproduction may also impose potentially severe bottlenecks for the transmission of their symbiotic microbes. For example, the life cycle of a typical trematode begins with a microscopic larva hatching from an egg to infect the first intermediate host (almost always a snail), in which it will multiply asexually (clonally) into a colony of sporocysts (Galaktionov and Dobrovolskij, 2003). The latter produce cercariae, genetically identical free-swimming infective stages that leave the snail to seek a second intermediate host (invertebrate or small vertebrate, depending on the species), in which they encyst as metacercariae to await ingestion by their definitive host (almost invariably a vertebrate). In this final host, the parasites quickly develop into mature worms which reproduce sexually and release eggs that usually reach the outside world in host feces. The persistence of a core microbiome, across these physiological events and huge changes in immediate environmental conditions experienced by small worms with limited powers of homeostasis, would be remarkable. Even if particular microbial taxa persist through the life cycle, their relative abundance may vary during ontogeny, as observed in the microbiomes of arthropod ectoparasites (Ben-Yosef et al., 2017; Ponnusamy et al., 2018). If the worm and its symbiotic microbes form an integrated functional unit consistent across generations, the holobiont (Dheilly, 2014; Bordenstein and Theis, 2015), their shared needs to counter host immune responses, feed and get transmitted will change from one life stage to the next. Selection may therefore have synchronized the proliferation of certain microbes playing different functional roles at distinct stages of the parasite life cycle.

These considerations lead to important questions, and in some cases, the biology of trematodes allows certain predictions. At what stage of the life cycle does the parasite microbiome peak in diversity? At what stage does the parasite microbiome comprise the most bacteria acquired from outside (i.e., the immediate host environment or the external environment)? We predict the answer to both questions to be the adult stage. In species without redial stages (like our model species; see below), only adult worms actively feed on host tissue by ingestion through a mouth; sporocysts and metacercariae feed passively by absorption through their tegument or cyst wall, whereas cercariae do not feed at all. This makes the adult intrinsically more likely to acquire bacteria (and least endosymbiotic ones) from the host. What are the greatest bottlenecks for the continuation of the bacterial community through the trematode life cycle? We expect the greatest reduction in bacterial diversity, i.e., the greatest loss of taxa, to occur at the egg stage linking the adult microbiome to that in sporocysts, in part because of the extremely small size of the egg (typically oval-shaped with a length < 100 μm). The mass production of cercariae by sporocysts may also lead to a reduction in microbiome diversity, as each cercaria probably harbors only a subset of the microbiome of its parent sporocyst. Is there a core trematode microbiome, consisting of bacteria occurring at relatively high prevalence among parasite individuals, possibly not found in the host or external environment, persisting through the whole life cycle and transmitted vertically across generations? If so, what taxa does it comprise, and are these associated with known functions? These are questions that can only be answered with data from culture-independent sequencing approaches.

Here, we address all above questions using the New Zealand freshwater trematode *Coitocaecum parvum* as a model organism. We characterize the microbiome of its sporocysts in snails, its free-swimming cercariae, its metacercariae in crustaceans, and its adults in fish. The intimate physical association of a parasite with its host poses challenges for the characterization of parasite microbiomes: host microbes may be attached to the parasite’s surface or ingested by the parasite. We performed a range of technical controls to account for potential contamination of parasite microbiomes by host microbes, and for each parasite analyzed we took samples from the corresponding host individual from which it was extracted to test for bacterial sharing between them. Our analysis determined the source of the bacteria in the microbiomes of each life stage, i.e., whether they originate from the previous life stage, the host tissues or the external environment. Importantly, we reveal the existence of a distinct core trematode microbiome, comprising several taxa that follow the parasite along its entire life cycle, impervious to the drastic changes in the host environments inhabited by the trematode. Our findings provide evidence that tiny parasitic worms contain diverse communities of symbiotic bacteria made up of a (more-or-less) permanent subset of taxa and also a life-stage dependent subset, suggesting possible ontogenetic shifts in the particular functional contribution of the microbiome to the host–parasite interaction.

## Materials and Methods

### Sample Collection, Processing and Metadata

A detailed version of the following methods is provided in Supplementary Information. Naturally infected *C. parvum* hosts spanning the parasite ontogenetic development were searched among snails (*Potamopyrgus antipodarum*), amphipods (*Paracalliope fluviatilis*), and fish (*Gobiomorphus cotidianus*) collected from Lake Waihola, South Island, New Zealand during the 2019 austral summer. Samples were collected under an approved Animal Use Protocol from the University of Otago (AUP-18-233). Immediately prior to animal collection, two types of environmental samples were collected with sterile cotton swabs, i.e., water (two samples) and lake sediment (two samples) Two controls for the swabs themselves were also taken by opening the swab and exposing it to natural air prior to saving it in a PowerBead Pro Tube. Environmental and control samples were snap frozen and kept in a −80°C freezer. Waihola lake water was also collected into sterile containers for maintenance of specimens in the laboratory until processing.

In the laboratory, snails were placed in individual sterile wells with lake water, and incubated for 2 days at 25°C under light to identify *C. parvum*-infected individuals through cercarial shedding. Amphipods were individually placed in sterile wells containing water and screened under the microscope for signs of infection (see Lagrue and Poulin, 2007). Fish were kept alive in aerated lake water until further processing.

All dissections were conducted in a sterile laminar flow cabinet, and between each sample tools were cleaned with bleach, and sterilized with ethanol and burning flame. Prior to dissections, two samples were taken with sterile swabs of the water in which each host species were kept, to serve as controls for contamination within the laboratory environment. Snails were brushed with sterile interdental brush in 99% EtOH, and rinsed thoroughly in heat-sterilized PBS prior to dissections. From the eight infected snails, we successfully isolated sporocysts (two per snail from eight snails, *n* = 16), cercariae (three per snail from three of the eight snails, *n* = 9, given the low cercarial output from this very small-bodied snail species) and snail tissue (adjacent to parasite tissue but free of it from five snails; *n* = 5). Amphipods were rinsed thoroughly in a series of 70%, and 99% EtOH, and then PBS. Metacercariae (1–3 per amphipod, *n* = 12) and amphipod tissue (whole body after parasite removal; *n* = 6) were collected. Fish were euthanized with an overdose of MS-222, and placed individually in sterile petri dishes. Before dissection, fish were brushed with Betadine (Sanofi) to prevent contamination of the body cavity with skin microbes. Their intestinal tract was aseptically removed from the abdominal cavity, and opened to find adult parasites. Adult worms (1–3 per fish, *n* = 10) and fish tissue (intestinal wall, clean of parasites and contents, *n* = 5) were collected.

Our operational definition of the parasite microbiome includes all microbes living inside the parasite’s body, and excludes those attached to the parasite surface. This may be conservative, but in the absence of information on the functional contribution of each microbe to the parasite’s biology, this definition avoids the erroneous inclusion of microbes that truly belong to the host or environmental microbiota. All tissue samples, both parasite and host, were cleaned from surface microbiota by vigorously pipetting up and down in PBS in sterile wells. Samples of the surface microbiota for each sample type was collected by pipeting 75 μl of the resulting ‘washing’ (two samples per host type and parasite life stage). For each host group a sample of the PBS solution was taken at the end of the procedures to account for any possible contamination of the solution. Samples were snap frozen and kept in a −80°C freezer until DNA isolation. Metadata on sample type (e.g., environmental, host type, parasite, controls), life stage (e.g., sporocyst, metacercaria), and host ID are given in Supplementary Table S1.

### Library Preparation and Microbiome Sequencing

DNA was extracted using the DNeasy PowerSoil Pro Kit (QIAGEN), following the manufacturer’s protocol, with modifications recommended for cells difficult to lyse by the Earth Microbiome Project (EMP) DNA Extraction Protocol (Marotz et al., 2017). Together with the isolated biological samples, two ZymoBIOMICS microbial community standards samples (MCS), and one reagent-only sample were also extracted to assess the performance and contamination of our workflow, respectively.

DNA libraries for each sample were prepared following EMP 16S Illumina Amplicon Protocol to amplify prokaryotes using paired-end community sequencing. The V4 hypervariable region of the prokaryotic bacterial 16S SSU rRNA gene was PCR-amplified and multiplexed using the universal bacterial primers 515F (Parada) – 806R (Apprill) (Apprill et al., 2015; Parada et al., 2016). Together with the biological samples of interest, one additional control sample of 0.2ng of the ZymoBIOMICS microbial community DNA standards (MCS DNA) and a reagent-only sample were also included. Samples were amplified in triplicate in a 20 μl mix composed of 5.6 μl of ultrapure water, 10 μl of MyFi^TM^ mix (Bioline), 8 μM of each primer and 2 μl of DNA template. The PCR conditions consisted of an initial denaturation step of 3 min at 95°C and 35 cycles, each consisting in one cycle of 45 s at 95°C, 60 s at 50°C, and 90 s at 72°C, followed by a final extension cycle of 10 min at 72°C. Triplicate libraries of each sample were pooled and run on a 2% agarose gel. We then used a quantitative binding approach to clean and normalize each amplicon with SequalPrep Kit (Invitrogen) following the manufacturer’s protocol. This protocol requires that DNA is present in excess (≥250 ng) for accurate normalization; given that several samples were below this requirement, we quantified DNA concentration with QuBit with 1X dsDNA HS Assay Kit (Invitrogen). Each amplicon library was then manually diluted to the lowest measured concentration of biological samples, and equal volumes of amplicons were combined in a single tube to construct the final libraries pool. The DNA concentration of this pool of libraries was quantified with QuBit (as above), and the average molecule length was determined using the Agilent 2100 bioanalyzer instrument (Agilent DNA 1000 Reagents). Combined barcoded libraries were sequenced on an Illumina MiSeq platform using the V2 reagent cartridge (250 bp, paired-end) through the Otago Genomics & Bioinformatics Facility (New Zealand).

### Sequence Processing

Data were received as demultiplexed paired-end raw sequences, and were processed and analyzed using the Quantitative Insights Into Microbial Ecology (QIIME) 2 software package (Bolyen et al., 2019). Adapters and primers were removed from raw sequences using the plugin *cutadapt* (with 0 error-rate and minimum length of 240 bp) (Martin, 2011), and quality filtered using the *dada2* plugin (Callahan et al., 2016) after inspection of quality profile plots of forward and reverse reads. The resulting amplicon sequence variants (ASVs) table was filtered to exclude non-bacterial, mitochondrial, chloroplast and ASVs without a phylum assignment, contaminants, and samples with low sequencing depth (i.e., frequency lower than 1,000, and/or with less than 8 ASVs) using the *feature-table* plugin (see Supplementary Information). A ‘reduced dataset’ which did not include ASVs not shared by at least two samples (*feature-table* plugin) was also created. For analyses regarding the diversity of parasite-associated microbial communities, these two datasets were further filtered to include only those samples extracted from parasite tissues. For each dataset, different taxonomic levels were assigned to the ASVs using the plugin *feature-classifier* (Bokulich et al., 2018) against the Greengenes 16S rRNA reference database (13_8 release) pre-trained on the 515F/806R region (Pedregosa et al., 2011).

Sequenced data quality was evaluated based on the observed composition and sequence quality of the ZymoBIOMICS microbial community standards (MCS and MCS DNA), against the expected data of these mock communities, using *quality-control* plugin. This analysis allowed us to assess how well our methods and pipeline estimate the microbial community present in the samples.

### Diversity Analyses

Diversity analyses were performed primarily using QIIME 2, and the R packages vegan (Oksanen et al., 2019) and phyloseq (McMurdie and Holmes, 2013) with default function settings unless otherwise noted. Prior to analyses, ASVs were aligned using the mafft program (Katoh et al., 2002) and used to construct a phylogenetic tree using the fasttree2 program (Price et al., 2010) with the *phylogeny* plugin. For analysis, the filtered ASVs and taxonomy tables, and the rooted tree were imported into R (R Core Team, 2018) with the qiime2R package (Bisanz, 2018) and together with the metadata combined into a phyloseq object. Given that one of the sources of potential ‘noise’ in metabarcoding analysis is the fine-scale data (here ASVs), analyses were also performed at the higher taxonomic ranks Phylum and Family using the agglomeration *phyloseq* function tax_glom. Phyloseq objects were evenly subsampled using *rarefy_even_depth()*.

We started by investigating the presence of a ‘core’ microbiome common to all parasite life stages, and specific to each life stage. First, Venn diagrams were created at the family level. We tested for the presence of a ‘core’ microbiome as defined by any taxon with a prevalence higher than 0.95, 0.75, or 0.50 with the *microbiome* package (Lahti and Shetty, 2012–2019) at different taxonomic levels. To infer which families had a higher relative abundance among life stages, we created heatmaps using *plot_ts_heatmap()* of the mctoolsr package (Leff, 2017). A tree plot was created over the full tree estimated from the alignment of parasite ASVs to visualize how microbiota components of the different life stages relate to each other, and how the life stages relate to each other.

The diversity within each parasite life stage (alpha diversity) was calculated using the following metrics: Faith’s phylogenetic diversity, evenness and Shannon diversity using the QIIME 2 *alpha-group-significance* plugin. The Kruskal–Wallis test was used to calculate pairwise comparisons between alpha diversity estimates among life stages.

To test whether life stages differ in community composition (beta diversity), we used phylogenetic-based indices which are useful even with low sequence coverage (Lemos et al., 2011), but also given that phylogenetic information is relevant to the questions in our study. Specifically, the qualitative unweighted Unifrac (Lozupone and Knight, 2005) and quantitative weighted UniFrac (Lozupone et al., 2007) distance metrics were calculated with *distance()*. First, to explore the structure of microbial communities, principal coordinates plots (PCoA) were created with *plot_ordination()* adding hulls as defined with *find_hull()*. Statistically significant differences among life stages were determined with permutational ANOVA performed with *adonis* and with multilevel pairwise comparisons with *pairwise.adonis()* with Benjamini and Hochberg’s (1995) (“BH-FDR”) correction for multiple testing with 9999 permutations. We further explored if there were differential abundances of bacterial phylotypes between consecutive life stages with DESeq2 (Love et al., 2014). DESeq() was called with default parameters, and results were contrasted by life stage, and an adjusted *p*-value cut-off of 0.05 was used for differences in relative abundances to be considered statistically significant.

### Sources of Parasite Microbiome

We tested whether the parasite’s microbial composition differed from that of its different hosts and environment using the same diversity analyses as described above. Using Venn diagrams, we determined if there were any taxa unique to the parasite bacterial community, irrespective of their abundances and prevalence.

To determine the likely main sources of each life stage microbiome, we used the Bayesian approach SourceTracker developed for R (Knights et al., 2011). For each parasite life stage (classified as ‘sink’), we used SourceTracker to estimate the proportion of bacteria originating from potential ‘sources’: environmental samples (water and sediment, and laboratory environment), the host, the prior parasite life stage, or unknown sources (representing one or more sources absent from the training data) using the ASV data of the reduced dataset with a rarefaction of 1,000. Samples were classified as sources (potential contributors to a given microbial community) or sinks (the community being investigated), and a total of four analyses were conducted (one per life stage). We then predicted for each sink and their respective sources training data, the proportional contribution of sources with *predict()*. Bar plots were created over the mean and standard deviation of the resulting proportion estimates of contributing sources for each parasite life stage, and also for the respective train data with ggplot2 (Wickham, 2016).

## Results

About 5.3 million demultiplexed paired-end raw sequences from 91 samples (including blank and control samples) were obtained. As outlined in the Methods, we analyzed two datasets: the main dataset, and a more conservative ‘reduced’ dataset which did not include ASVs not shared by at least two samples. After sequence quality control filtering (see Supplementary Information), the main and reduced datasets consisted of 60 samples with 2,648 ASVs, and 49 samples (since 11 had frequency below 1,000) with 937 ASVs, respectively. Microbial communities from parasite specimens included 30 samples with 360 ASVs, and 22 samples with 70 ASVs for the main and reduced datasets, respectively. Taxonomic assessment based on the Greengenes 16S rRNA reference database revealed biased classification below the family level (Supplementary Figure S1b). Therefore, classification below family level (when given) should be interpreted with caution.

### Parasite ‘Core’ Microbiome

**FIGURE 1:**
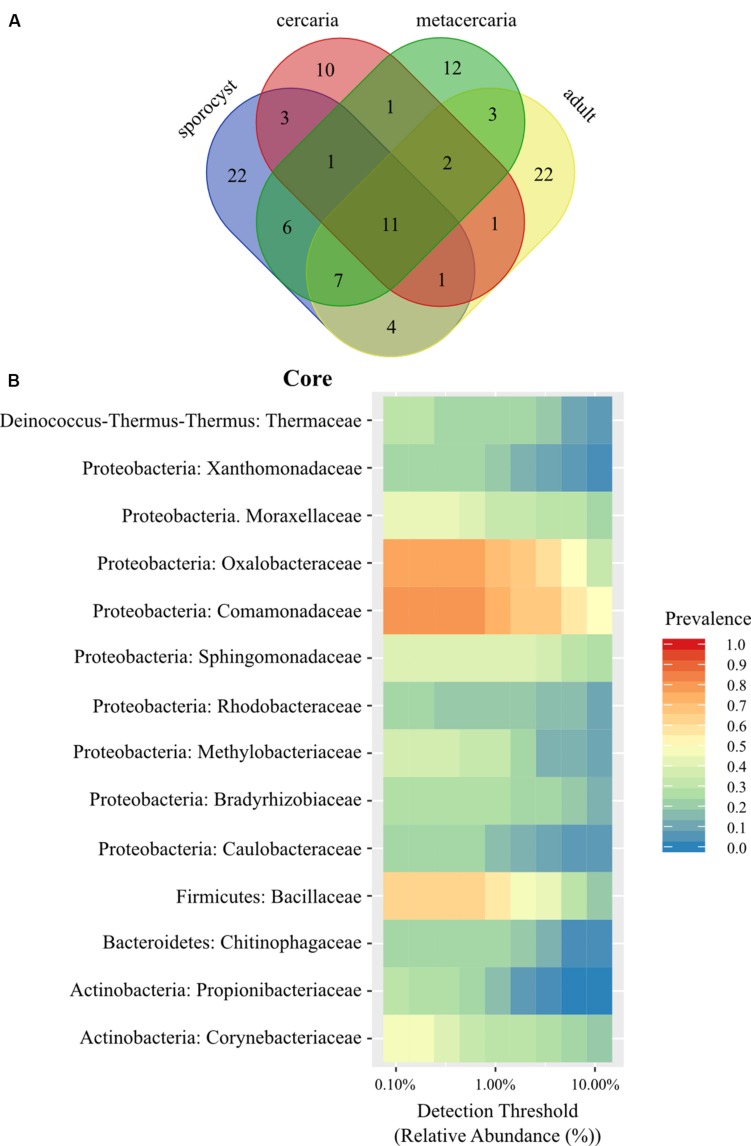
**(A)** Venn diagram showing the number of shared bacterial families among life stages of the trematode parasite *Coitocaecum parvum*. **(B)** Heat map of the bacterial phylogenetic core of *C. parvum* at family level (within respective phylum) as a function of the abundance threshold for taxa with prevalence above 0.2. The *x*-axis represents the detection thresholds (indicated as relative abundance) from lower (left) to higher (right) abundance values. Color shading indicates the prevalence of each bacterial family among samples for each abundance threshold. As we increase the detection threshold, the prevalence decreases.

The taxonomic composition of microbial communities in *C. parvum* life stages includes taxa from the Bacteria domain belonging to 16 phyla, 37 classes, 58 orders, and 102 families. The family level Venn diagram shows that 11 families were shared across all parasite life stages, and that several were unique to each life stage (Figure 1A and Supplementary Table S2). However, no ASV occurred in all parasite samples across all life stages, and at a prevalence higher than 0.5. The maximum threshold prevalence for detection of ASVs shared by all life stages was 0.3, represented by *Geobacillus vulcani* (Bacillaceae) and *Variovorax* (Comamonadaceae). When evaluating prevalence at a higher taxonomic rank, i.e., species, one species, *Ralstonia* sp. (Oxalobacteraceae), was detected at a prevalence threshold of 0.5 and shared by all life stages. At the genus level, the two genera *Geobacillus* (Bacillaceae) and *Ralstonia* (Oxalobacteraceae) were detected at a prevalence threshold of 0.5 and shared by all life stages. At the family level, two families occurred among all life stages at the higher prevalence threshold of 0.75 (Comamonadaceae and Oxalobacteracea), and one additional family (Bacillaceae) could be included in the parasite family level ‘core’ with a prevalence threshold of 0.50 (Figure 1B) (see Supplementary Information for results pertaining to the reduced dataset).

Life stage-specific ‘core’ analysis revealed higher prevalence values at a finer scale (i.e., ASVs), between 0.75 and 0.5 for all life stages in the full dataset with the exception of cercariae [one for sporocysts (Thermaceae), one for metacercariae (Bacillaceae), and one for adult worms (Comamonadaceae)]. While these taxa had higher prevalence, none was found to be significantly associated with a particular life stage when taking into account relative abundances (all cases *p* > 0.05). To determine if any of these taxa was exclusive to the parasite, a second family level Venn diagram was created including all other samples. Twenty families were identified as unique to the parasite (Supplementary Table S3), but not including any of the families identified above with prevalences above 0.50. When analyzing by life stage, one (Streptococcaceae) of these 20 families occurred in all life stages, two were exclusive to the adults and sporocysts (Enterococcaceae, Staphylococcaceae), seven exclusive to sporocysts, four to metacercariae and six to the adult worms. Venn lists also highlighted some sharing of bacterial taxa between a parasite life stage and the host of a different life stage (e.g., Ruminococcaceae, a main component of bacterial communities in adult worms, is shared with snails and the environment). This is likely caused by variation in microbial composition among samples (alpha diversity) and small sample sizes.

### Community Ecology

**FIGURE 2:**
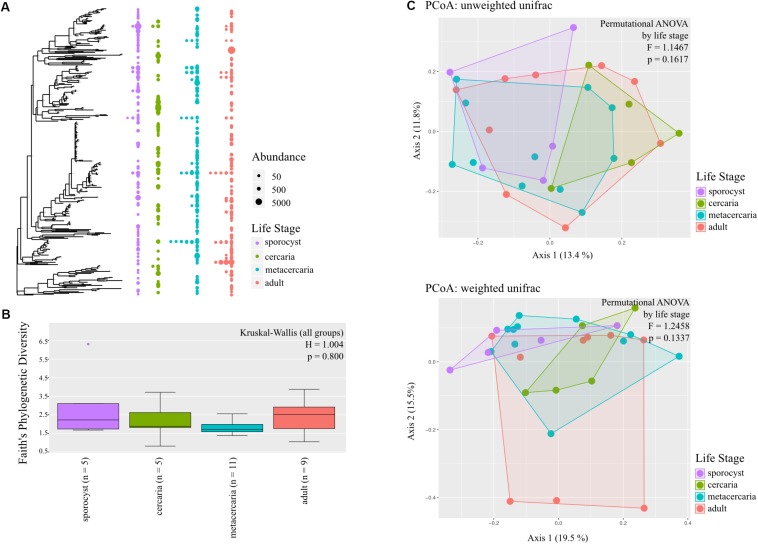
Bacterial community composition of the different life stages of the trematode parasite *Coitocaecum parvum*. **(A)** Annotated phylogenetic tree showing the relationships among taxa (amplicon sequence variants) making up the bacterial communities in the four parasite life stages. Dots are shown when a taxon is observed in the different life stages, with diameter proportional to abundance (read count) in a given sample. Samples are merged into the same life stage column unless the taxon is shared by more than one sample. **(B)** Box plots showing Faith’s phylogenetic diversity measure of bacterial community richness for each life stage; result of Kruskal–Wallis (all groups) test also shown. **(C)** Principal coordinates analyses ordinations based on unweighted and weighted unifrac distance matrices, with hulls delimiting each life stage group of samples; results of permutational ANOVA test are also shown.

The annotated phylogenetic tree shows that only few ASVs are shared by many samples, but they are closely related within each life stage, without a clear distinction in diversity level among life stages (Figure 2A). Microbial diversity did not differ significantly among life stages for any of the metrics used (in all cases *p* > 0.1, Figure 2B and Supplementary Table S4), and for any of the taxonomic levels investigated. *Post hoc* pairwise comparisons show that, for the reduced dataset only, microbial communities of adult worms had higher Faith’s phylogenetic diversity than those of metacercaria (Kuskal–Wallis pairwise *H* = 5.357, df = 1; *p* = 0.021, BH-FDR = 0.124), and that metacercariae had higher evenness than sporocysts (Kuskal–Wallis pairwise *H* = 4.167, df = 1; *p* = 0.041, BH-FDR = 0.247).

Testing for changes in community composition along the parasite ontogenetic development with principal coordinate analysis on unweighted and weighted Unifrac distances showed very little clustering among life stage, with only a fraction of variation explained by the first two axes (Figure 2C). Permutational ANOVA on the different distance metrics further supported these observations, and parasite life stage was not found to predict parasite microbial community structure at any taxonomic level and in either the full or reduced datasets (all metrics *p* > 0.05, Supplementary Table S5). However, *post hoc* tests identified cercariae and metacercariae as significantly different from each other based on unweighted unifrac (*F* = 1.579, *p* = 0.0313, but BH-FDR = 0.188; similar for estimates at family level). However, for the reduced dataset this was not the case and instead adults and metacercariae presented significantly different communities based on unweighted unifrac (*F* = 1.751, *p* = 0.038, BH-FDR = 0.229; same for phylum and family level). Analysis of homogeneity of dispersion showed that all groups presented similar dispersions (all tests *p* > 0.05). Knowing that there was no significant structure in microbial community of the parasite driven by life stage, we estimated whether any taxa were differentially abundant across the four life stages. The results revealed only very few taxa showing differential abundance from one life stage to the next (see Supplementary Information).

### Source of Parasite Microbiome

Microbial diversity differed significantly among sample types (in all cases *p* < 0.01, Supplementary Table S6). Pairwise comparisons revealed that microbial communities from the parasite had significantly lower Faith’s phylogenetic diversity and Shannon diversity than environmental samples (for both Faith’s and Shannon: *H* = 10.286, *p* = 0.001, BH-FDR = 0.009), but there were no significant differences in evenness (*p* > 0.05). Parasite samples presented higher estimates for Shannon diversity than those of fish and snail host tissue (vs. snail: *H* = 7.476, *p* = 0.006, BH-FDR = 0.016; vs. fish: *H* = 5.455, p = 0.020, BH-FDR = 0.029), but lower estimates of Faith’s phylogenetic diversity and Shannon diversity than the amphipod host (Faith’s: *H* = 13.638, *p* = 0.000 BH-FDR = 0.002; Shannon: *H* = 13.018, *p* = 0.000, BH-FDR = 0.003). When analyzing by life stage, microbial communities in sporocysts had higher evenness and Shannon community richness than their snail host, cercariae also have higher evenness than snail hosts; metacercariae have significantly lower Faith’s phylogenetic diversity and Shannon estimates but higher evenness than their amphipod hosts; and adult worms have higher Shannon estimates than their fish host (Supplementary Table S6, but in all cases BH-FDR > 0.05). At family level, only metacercariae had significantly less phylogenetically diverse bacteria than their hosts (*p* = 0.015).

**FIGURE 3:**
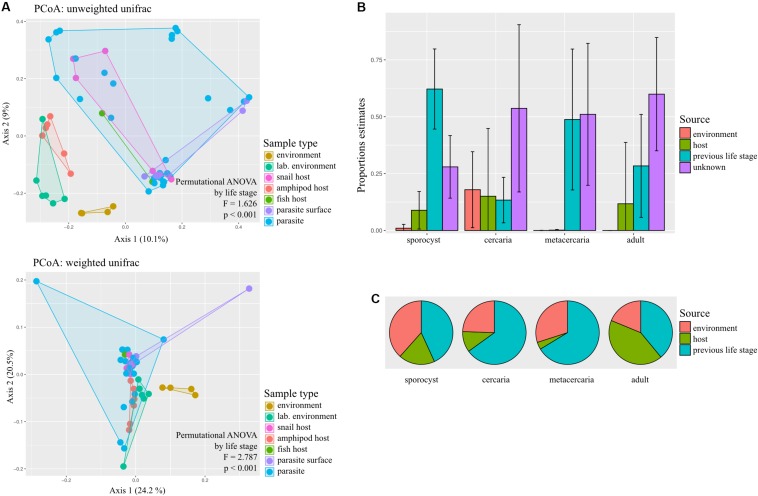
Sources of bacterial communities in the trematode parasite *Coitocaecum parvum*. **(A)** Principal coordinates analyses ordinations based on unweighted and weighted unifrac distance matrices, with hulls delimiting each group of samples; results of permutational ANOVA test are also shown. The sample types ‘parasite’ and ‘parasite surface’ comprise data pooled across life stages. **(B)** Histograms representing the average and standard deviation of the relative contribution of each potential source of the microbial communities of each parasite life stage. **(C)** Pie charts showing for each parasite life stage the average proportion of times that each respective source was incorrectly classified as unknown during SourceTracker training (for complete results of SourceTracker training, see Supplementary Figure S3). The proportion of incorrect assignments for a source may be the main contributor of the ‘unknown’ source in **(B)**, as opposed to an unsampled source excluded from the training data.

Principal coordinate analysis on unweighted and weighted Unifrac distances showed different degree of community similarity (or dissimilarity) among samples (Figure 3A). When considering only what is present in the community (unweighted unifrac), we uncovered distinct clusters among sample types; environmental samples, lab environment and amphipods seem to have a distinct microbiota from that of the parasite, but not snail and fish hosts. However, when considering relative abundance of bacterial taxa, only the environment seems to have a distinct microbial community (although for the reduced dataset, PCoA on weighted unifrac shows that all main sample types present distinct microbial communities; Supplementary Figure S2). Permutational ANOVA indicated significant differences in microbial community among samples (unweighted unifrac: *F* = 1.6256, *p* < 0.001; weighted unifrac: *F* = 2.787, *p* < 0.001). However, in some cases, groups presented different dispersions (unweighted unifrac: *F* = 10.429, *p* < 0.001; unweighted unifrac: *F* = 1.8311, *p* = 0.0903; for reduced dataset both tests *p* > 0.05). *Post hoc* pairwise analysis indicated that the microbial community of each parasite life stage differs significantly from that of the environment (*p* < 0.05, and several cases BH-FDR < 0.05, Table 1). With respect to their respective hosts (their direct environment), the composition of the parasite microbiota at each life stage did not differ significantly from their hosts (unweighted unifrac *p* > 0.05, similar across taxonomic levels), the exception being metacercariae which differed from their amphipod host (*F* = 2.986, *p* = 0.000, BH-FDR = 0.007, consistency across both datasets and taxonomic levels, Table 1 and Supplementary Table S7). However, when relative abundance is considered in addition to taxonomic composition (weighted unifrac), all parasite life stages were found to harbor significantly different communities from their respective hosts, the only exception being for adult worms (Table 1, but results depend on the taxonomic level considered).

**TABLE 1:** Permutational ANOVA pairwise tests of beta diversity comparisons between the four life stages of the trematode parasite *Coitocaecum parvum* and potential external sources (their respective hosts, their surface microbiota, and the environment) with raw *p*-values and *p*-values corrected for multiple testing (BH-FDR, Benjamini and Hochberg correction).

			Unweighted-unifrac			Weighted-unifrac			Unweighted-unifrac-by Phylum			Unweighted-unifrac-by Family		
				
		Sample size	pseudo-F	*p*-value	BH-FDR	pseudo-F	*p*-value	BH-FDR	pseudo-F	*p*-value	BH-FDR	pseudo-F	*p*-value	BH-FDR
Host	Life Stage													
Snail	Sporocyst	10	1.079	0.322	0.390	2.390	**0.015**	0.064	0.566	0.694	0.798	1.025	0.441	0.507
Snail	Cercaria	10	1.146	0.211	0.314	2.484	**0.015**	0.064	1.384	0.167	0.329	1.907	**0.007**	**0.039**
Amphipod	Metacercaria	17	2.986	**0.000**	**0.007**	4.256	**0.005**	0.051	7.011	**0.001**	**0.014**	6.245	**0.000**	**0.007**
Fish	Adult	11	1.151	0.226	0.322	2.067	0.183	0.288	1.428	0.180	0.329	2.220	**0.020**	0.055
Surface microbiota	Life Stage													
sporPBS	Sporocyst	7	1.197	0.190	0.302	0.345	0.810	0.887	1.368	0.286	0.387	1.228	0.143	0.199
mPBS	Metacercaria	14	1.054	0.373	0.429	0.354	0.890	0.912	0.147	0.900	0.920	0.475	0.973	0.973
adPBS	Adult	11	0.955	0.467	0.499	2.729	0.142	0.243	1.514	0.186	0.329	1.549	0.071	0.120
Environment	Life Stage													
SLabEnv	Sporocyst	7	1.661	**0.048**	0.112	1.669	0.238	0.332	3.106	0.095	0.228	2.877	**0.048**	0.088
Environment	Sporocyst	9	2.389	**0.007**	**0.044**	5.595	**0.007**	0.051	8.195	**0.006**	0.058	5.252	**0.007**	**0.039**
SLabEnv	Cercaria	7	1.453	**0.048**	0.112	3.371	**0.048**	0.104	2.666	**0.048**	0.183	2.914	**0.048**	0.088
Environment	Cercaria	9	2.159	**0.008**	**0.044**	7.285	**0.008**	0.054	9.354	**0.008**	0.064	5.942	**0.008**	**0.039**
ALabEnv	Metacercaria	13	1.501	0.061	0.134	3.003	0.051	0.107	4.252	**0.014**	0.066	2.812	**0.012**	**0.048**
Environment	Metacercaria	15	2.814	**0.001**	**0.010**	10.320	**0.001**	**0.016**	16.214	**0.001**	**0.014**	7.793	**0.001**	**0.010**
FlabEnv	Adult	11	1.802	**0.019**	0.079	2.311	0.198	0.288	3.218	0.056	0.191	3.074	**0.019**	0.054
Environment	Adult	13	2.361	**0.001**	**0.010**	6.032	**0.001**	**0.021**	11.674	**0.002**	**0.024**	6.695	**0.001**	**0.010**

*spor, sporocyst; m, metacercaria; ad, adult; SLabEnv, water in which snails were kept in the lab; ALabEnv: water in which amphipods were kept in the lab; FLabEnv: water in which fish were kept in the lab. Significant *p*-values are shown in bold.*

Using SourceTracker, we aimed to identify the main sources of microbiota of each parasite life stage. Results indicate that the previous life stage was the main known source of bacteria for all life stages, with the current host and the environment making much lower contributions (Figure 3B). To determine if the unknown source was actually an artifact of rarefaction, we also analyzed the proportion of each source that was correctly identified during the training step of the analysis (Figure 3C; see Supplementary Figure S3 for full results). Of all possible sources, the previous life stage was the most frequently incorrectly assigned to ‘unknown’ (Figure 3C). Sporocysts had the lowest proportion of unknown source (∼0.25); also, most sources appear to have been correctly classified. For the cercariae, which had a 0.50 proportion of unknown source, this may in fact be attributed to the previous life stage (i.e., sporocyst) which was incorrectly identified as ‘unknown’ very frequently during training. This may also be the case for the metacercariae. For adult worms, both the current host (fish) and previous life stage (metacercaria) were frequently incorrectly classified as unknown, so is unclear which may have a bigger contribution.

## Discussion

Mounting evidence supports the view that metazoans and their microbial symbionts form integrated entities, or holobionts, which may represent true evolutionary units (Gilbert et al., 2012; McFall-Ngai et al., 2013; Bordenstein and Theis, 2015). Accordingly, natural selection at the holobiont level could favor traits that are costly to microbes if they benefit the animal in which they reside (Zilber-Rosenberg and Rosenberg, 2008). Selection at the holobiont level is possible whether microbes are horizontally or vertically transmitted (Roughgarden et al., 2018). A recent mathematical model suggests that this is more likely under two conditions: microbes must be predominantly vertically transmitted, and the animal harboring them must have a short generation time (Van Vliet and Doebeli, 2019). Parasites and their microbiome are ideal candidates to meet these conditions, and are therefore great model systems for advancing the holobiont concept. Here, we provide evidence that a trematode parasite possesses a phylogenetically diverse microbiome, distinct from that of its hosts or the external environment, consisting both of taxa specific to each life stage in each of the parasite’s different host species, as well as a small core of bacterial taxa that persistently occur across the parasite’s life stages.

Our evidence for a core microbiome requires a few words of caution. Firstly, it must be pointed out that a true core microbiome would manifest as the exact same ASVs, or within species-level variation, at each life stage and across all samples. Our findings identify a few shared ASVs but only at lower prevalence. However, above the ASV level, we found bacteria present across all life stages at a high enough prevalence to consider them core microbiota. The number of taxa included in the core microbiome increased with the level of taxonomy, but nevertheless comprised only few taxa. This may be due to a range of factors, including detection limits for ASVs with low abundance at certain life stages, or limited sample size of individual parasites screened at each life stage. Secondly, whether the same set of bacterial taxa are also found in other populations of the trematode *Coitocaecum parvum*, i.e., whether they represent a true species-level core microbiome as opposed to a population-specific one, remains to be determined. Furthermore, formal demonstration of vertical transmission would require following a single parasite cohort across generations under laboratory conditions. However, because certain bacterial taxa were found in all parasite life stages, but not in host tissues nor in the external environment, we propose that the parasite’s core microbiome is vertically transmitted, as this mode of transmission provides the most parsimonious explanation (if not the only explanation) for our findings.

We have taken steps to avoid the pitfalls potentially associated with the analysis of 16S rRNA sequences for microbial diversity studies. Depending on alpha diversity (number of taxa) per sample, many samples may be required to achieve a representative characterization of microbiomes (Lemos et al., 2011). Our sample sizes per life stage are modest, which may result in significant differences in microbiome composition when there really is none. However, our focus was not on differences, but rather on similarities in microbiome composition among parasite life stages; small sample sizes are much less likely to matter in this case. Furthermore, we also characterized the microbiome of each individual host from which we extracted the parasites studied here; therefore, bacteria found exclusively in the parasites but not in the particular host individuals from which they came (and not found in the external environment), provide solid evidence for the existence of a core parasite microbiome. In addition, our samples generally had modest diversity, ASVs with low sequencing depth were excluded, and we used measures of phylogenetic diversity in addition to basic estimates of compositional diversity, all factors that should reduce the risk of biased results (Lemos et al., 2011). We also reduced bias potentially associated with our workflow by using standard mock communities to test the robustness of our methods to detect bacterial taxa (see Supplementary Table S8), and used a range of other quality control procedures to eliminate contaminants and other sources of error. Finally, we repeated the analyses on a ‘reduced’ dataset, which included only ASVs detected in at least two samples, and thus provided a more conservative test of our predictions based on more stringent criteria for data inclusion. Overall, we are confident that the patterns we observed are real ones.

We expected the diversity of the trematode microbiome to peak at the adult stage, not simply because it has the largest body size, but because it is the only life cycle stage in the trematode studied here that actively feeds by ingesting host tissues, providing an invasion opportunity for host bacteria to colonize the parasite. In fact, our results reveal no consistent difference in microbiome diversity among life stages, across the different diversity metrics used, the different taxonomic levels analyzed, and the two different datasets (full and reduced). Adult worms also did not have microbiomes that were more similar in terms of diversity to those of their fish hosts than other life stages compared with their respective hosts. However, our results demonstrate that at the sporocyst, cercarial and metacercarial life stages, when considering both the composition and relative abundance of microbial taxa, the parasite’s microbiome differs from that of the host, but not at the adult stage. This might indicate that consumption of host tissues may lead to the partial homogenization of host and trematode microbiomes, by allowing entry of host microbes and their establishment into the parasite.

Although our analyses uncovered microbial taxa that were unique to the parasite, or to particular life stages of the parasite, these generally occurred at low to moderate prevalence (i.e., in a small to moderate proportion of individual parasites analyzed). The fact that these are not present in every single individual does not mean that they do not represent a core microbiome. Firstly, for basic statistical reasons, the prevalence values we obtained are likely underestimates of true prevalence due to our small sample sizes (Gregory and Blackburn, 1991). Secondly, they may also reflect the expected inter-individual variation in microbiome composition that would result from imperfect ontogenetic or vertical transmission. The well-studied bacterial taxon *Neorickettsia*, a common symbiont of trematode parasites (Vaughan et al., 2012), provides a good illustration. Several *Neorickettsia* genotypes have been reported from different life stages of multiple trematode species from around the world, although the bacteria never reach 100% prevalence (Vaughan et al., 2012; Greiman et al., 2014). In a study of natural infections of the trematode *Plagiorchis elegans* in its snail hosts, even if practically all sporocysts within a snail harbored the symbiont *Neorickettsia*, the latter suffered a transmission bottleneck during the asexual production of clonal cercariae, as only between 11 and 91% of cercariae leaving a snail host carried *Neorickettsia* (Greiman et al., 2013). Higher transmission rates are possible under ideal laboratory conditions, though still with substantial variation in the abundance of *Neorickettsia* acquired by each clonal cercaria (Greiman and Tkach, 2016). Nevertheless, the frequent loss of this one symbiont among cercariae all issued from the same clonal colony of sporocysts under natural conditions suggests that the core microbiomes of trematodes must vary among individuals: if other bacterial taxa are equally imperfect in their vertical transmission, then most microbial taxa will not occur in every parasite individual.

Despite the transmission bottleneck at the sporocyst-to-cercaria transition (and possibly also during egg production by adult worms), the trematode microbiome retained its distinctive signature throughout the life cycle. The diversity of the microbiome did not vary significantly among life stages, nor did its taxonomic composition. The relative abundance of certain component taxa changed during the ontogeny of the parasite, with different microbes peaking in abundance at different life stages, but this pattern was limited to very few taxa. Our analysis of the origins of the microbiome of each parasite life stage demonstrated that the previous life stage was consistently the main source of microbial taxa, compared to other potential sources (host, external environment including lake water and sediment), even given the uncertainty in quantifying the contribution of each source. Overall, there is consistency across the different analyses we performed on our data (PCoA on weighted and unweighted unifrac distances, permutational ANOVAs, SourceTracker) to indicate the persistence of the microbiome through the life cycle, although with life stage-specific components as well. We found that 11 bacterial families are shared across all parasite life stages. Perhaps more importantly, 20 bacterial families, even if each of them is not observed in every life stage, were found uniquely in the parasite’s microbiome, and not in their respective hosts or environment. One of them, Streptococcaceae occurred across all life stages, while two other families (Enterococcaceae, Staphylococcaceae) were found in two consecutive life stages, adults and sporocysts. These may play functional roles in the holobiont, i.e., the integrated trematode-plus-microbiome unit. Streptococcaceae was represented only by *Streptococcus* spp. These are considered to be either opportunistic pathogens or commensal bacteria, performing primary fermentation, although some strains can modulate toxins (Vitetta et al., 2019) or even have immunomodulatory properties (van den Bogert et al., 2014). Enterococcaceae and Staphylococcaceae were represented by *Enterococcus* spp. and *Staphylococcus* spp., respectively. Both taxa are facultative anaerobes, also involved in fermentative processes. These taxa may perform functions primarily related to host nutrient metabolism, with Streptococci also potentially contributing to the parasite’s immune homeostasis. It is however worth mentioning that *Streptococcus*, *Enterococcus*, and *Staphylococcus* have previously been identified in negative controls of other studies (Eisenhofer et al., 2019). Nevertheless, all these taxa have been recorded from tissues of parasitic nematodes, with the presence of *Streptococcus* visually confirmed by fluorescent *in situ* hybridization (Sinnathamby et al., 2018). *Enterococcus* and *Staphylococcus* have also been found in the bacteriome of parasitic fly larvae (Ben-Yosef et al., 2017). As described above, our framework and stringent criteria for data inclusion (and exclusion) allow us some confidence regarding the inclusion of these taxa as real components of the trematode microbiome.

Microbiomes are powerful modifiers of animal phenotypes (Lynch and Hsiao, 2019). Small differences in microbiomes have been linked to marked intraspecific variation in physiology, morphology or behavior (e.g., Kapheim et al., 2015; Leclair et al., 2016; Takacs-Vesbach et al., 2016). For example, the presence of particular microbes in a parasite’s microbiome can determine whether or not an individual parasite can manipulate its host’s behavior (Dheilly et al., 2015b). In our model species *Coitocaecum parvum*, metacercariae can adopt one of two distinct developmental pathways: most individuals remain small and await transmission to a fish definitive host before completing their development, whereas a smaller proportion display progenesis, i.e., accelerated growth, precocious maturation and reproduction within the amphipod intermediate host without the need to transfer to a fish host (Poulin, 2003; Lagrue and Poulin, 2007). The presence or absence of fish odors in the water has an effect on whether metacercariae adopt the normal or progenetic pathway, but only explains some of the variance in mode of development among individuals (Poulin, 2003; Lagrue and Poulin, 2007). Is the microbiome of individual metacercariae also influencing which developmental route they follow? In the present study, four bacterial families were found exclusively in metacercariae (Aerococcaceae, Dermacoccaceae, Brucellaceae, and an unclassified Lactobacillales), and not in other life stages, nor in hosts or the environment. Is the presence/absence of one or more of these bacterial taxa necessary for progenesis? These are questions that can be tackled with more extensive sequencing of the microbiomes of metacercariae, possibly combined with targeted experimental manipulation of bacterial communities within metacercariae. For instance, either transplanting microbes into microbe-free trematodes, or knocking out targeted bacteria with antibiotic treatments, although logistically challenging, would confirm the functional roles, if any, played by the microbiome in the parasite’s development. These are also questions that highlight the potentially huge role of microbiomes in parasite biology, and stress the importance of incorporating parasite microbiomes in future investigations of host-parasite interactions.

## Data Availability Statement

All microbial 16S rRNA sequences were deposited in the Sequence Read Archive (SRA, https://www.ncbi.nlm.nih.gov/sra/) under accession number PRJNA623422. Metadata, raw data and scripts used in this study are available on the OSF repository (https:/ /osf.io/pmq7r/?view_only=bca6433734c246bab3da0dc24 3ff4706).

## Ethics Statement

The animal study was reviewed and approved by University of Otago Animal Ethics Committee, permit # AUP-18-233.

## Author Contributions

RP, ND, and FJ designed the study. FJ obtained and processed the samples, and conducted all genetic, bioinformatic and statistical analyses, with input from ND. FJ and RP co-wrote the manuscript, with input from ND.

## Conflict of Interest

The authors declare that the research was conducted in the absence of any commercial or financial relationships that could be construed as a potential conflict of interest.

## References

[B1] Apprill A. McNally S. Parsons R. Weber L. (2015). Minor revision to V4 region SSU rRNA 806R gene primer greatly increases detection of SAR11 bacterioplankton. *Aquat. Microb. Ecol.* 75 129–137.

[B2] Benjamini Y. Hochberg Y. (1995). Controlling the false discovery rate: a practical and powerful approach to multiple testing. *J. Roy. Stat. Soc. B* 57 289–300.

[B3] Ben-Yosef M. Zaada D. S. Y. Dudaniec R. Y. Pasternak Z. Jurkevitch E. Smith R. J. (2017). Host-specific associations affect the microbiome of *Philornis downsi*, an introduced parasite to the Galápagos Islands. *Mol. Ecol.* 26 4644–4656. 10.1111/mec.14219 28664982

[B4] Bisanz J. E. (2018). *Qiime2r**: Importing Qiime2 Artifacts And Associated Data Into R Sessions.* Available online at: https://github.com/jbisanz/qiime2R (accessed September 21, 2019).

[B5] Bokulich N. A. Kaehler B. D. Rideout J. R. Dillon M. Bolyen E. Knight R. (2018). Optimizing taxonomic classification of marker-gene amplicon sequences with QIIME 2’s q2-feature-classifier plugin. *Microbiome* 6:90. 10.1186/s40168-018-0470-z 29773078 PMC5956843

[B6] Bolyen E. Rideout J. R. Dillon M. R. Bokulich N. A. Abnet C. C. Al-Ghalith G. A. (2019). Reproducible, interactive, scalable and extensible microbiome data science using QIIME 2. *Nat. Biotechnol.* 37 852–857. 10.1038/s41587-019-0252-6 31341288 PMC7015180

[B7] Bordenstein S. R. Theis K. R. (2015). Host biology in light of the microbiome: ten principles of holobionts and hologenomes. *PLoS Biol.* 13:e1002226. 10.1371/journal.pbio.1002226 26284777 PMC4540581

[B8] Bouchery T. Lefoulon E. Karadjian G. Nieguitsila A. Martin C. (2013). The symbiotic role of *Wolbachia* in *Onchocercidae* and its impact on filariasis. *Clin. Microbiol. Infect.* 19 131–140. 10.1111/1469-0691.12069 23398406

[B9] Callahan B. J. McMurdie P. J. Rosen M. J. Han A. W. Johnson A. J. A. Holmes S. P. (2016). DADA2: high-resolution sample inference from Illumina amplicon data. *Nat. Methods* 13 581–583. 10.1038/nmeth.3869 27214047 PMC4927377

[B10] Castiglioni P. Hartley M.-A. Rossi M. Prevel F. Desponds C. Utzschneider D. T. (2017). Exacerbated leishmaniasis caused by a viral endosymbiont can be prevented by immunization with its viral capsid. *PLoS Negl. Trop. Dis.* 11:e0005240 10.1371/journal.pbio.1005240 PMC5242429 28099431

[B11] Dheilly N. M. (2014). Holobiont-holobiont interactions: redefining host-parasite interactions. *PLoS Pathog.* 10:e1004093 10.1371/journal.pbio.1004093 PMC4081813 24992663

[B12] Dheilly N. M. Bolnick D. Bordenstein S. Brindley P. J. Figueres C. Holmes E. C. (2017). The parasite microbiome project: systematic investigation of microbiome dynamics within and across parasite-host interactions. *mSystems* 2:e050-17. 10.1128/mSystems.00050-17 28761932 PMC5516220

[B13] Dheilly N. M. Martinez J. M. Rosario K. Brindley P. J. Fichorova R. N. Kaye J. Z. (2019). Parasite microbiome project: grand challenges. *PLoS Pathog.* 15:e1008028 10.1371/journal.pbio.1008020 PMC6786532 31600339

[B14] Dheilly N. M. Maure F. Ravallec M. Galinier R. Doyon J. Duval D. (2015b). Who is the puppet master? Replication of a parasitic wasp-associated virus correlates with host behaviour manipulation. *Proc. Roy. Soc. B* 282:20142773. 10.1098/rspb.2014.2773 25673681 PMC4345448

[B15] Dheilly N. M. Poulin R. Thomas F. (2015a). Biological warfare: microorganisms as drivers of host-parasite interactions. *Infect. Genet. Evol.* 34 251–259. 10.1016/j.meegid.2015.05.027 26026593

[B16] Diaz Heijtz R. Wang S. Anuar F. Qian Y. Björkholm B. Samuelsson A. (2011). Normal gut microbiota modulates brain development and behavior. *Proc. Nat. Acad. Sci. U.S.A.* 108 3047–3052. 10.1073/pnas.1010529108 21282636 PMC3041077

[B17] Eisenhofer R. Minich J. J. Marotz C. Cooper A. Knight R. Weyrich L. S. (2019). Contamination in low microbial biomass microbiome studies: issues and recommendations. *Trends Microbiol.* 27 105–117. 10.1016/j.tim.2018.11.003 30497919

[B18] Ezenwa V. O. Gerardo N. M. Inouye D. W. Medina M. Xavier J. B. (2012). Animal behavior and the microbiome. *Science* 338 198–199. 23066064 10.1126/science.1227412

[B19] Faith J. J. Guruge J. L. Charbonneau M. Subramanian S. Seedorf H. Goodman A. L. (2013). The long-term stability of the human gut microbiota. *Science* 341 1237439. 10.1126/science.1237439 23828941 PMC3791589

[B20] Feldhaar H. (2011). Bacterial symbionts as mediators of ecologically important traits of insect hosts. *Ecol. Entomol.* 36 533–543. 10.1007/s00248-016-0829-2 27543560

[B21] Galaktionov K. V. Dobrovolskij A. A. (2003). *The Biology and Evolution of Trematodes.* Dordrecht: Kluwer Academic Publishers.

[B22] Gilbert S. F. Sapp J. Tauber A. I. (2012). A symbiotic view of life: we have never been individuals. *Q. Rev. Biol.* 87 325–341. 10.1086/668166 23397797

[B23] Gregory R. D. Blackburn T. M. (1991). Parasite prevalence and host sample size. *Parasitol. Today* 7 316–318. 10.1016/0169-4758(91)90269-t 15463402

[B24] Greiman S. E. Tkach V. V. (2016). The numbers game: quantitative analysis of *Neorickettsia* sp. propagation through the complex life cycle of its digenean host using realtime qPCR. *Parasitol. Res.* 115 2779–2788. 10.1007/s00436-016-5027-0 27041341

[B25] Greiman S. E. Tkach V. V. Pulis E. Fayton T. J. Curran S. S. (2014). Large scale screening of digeneans for *Neorickettsia* endosymbionts using real-time PCR reveals new *Neorickettsia* genotypes, host associations and geographic records. *PLoS One* 9:e98453 10.1371/journal.pbio.10098453 PMC4049572 24911315

[B26] Greiman S. E. Tkach V. V. Vaughan J. A. (2013). Transmission rates of the bacterial endosymbiont, *Neorickettsia risticii*, during the asexual reproduction phase of its digenean host, *Plagiorchis elegans*, within naturally infected lymnaeid snails. *Parasit. Vect.* 6:303. 10.1186/1756-3305-6-303 24383453 PMC3924192

[B27] Hooper L. V. Littman D. R. Macpherson A. J. (2012). Interactions between the microbiota and the immune system. *Science* 336 1268–1273. 22674334 10.1126/science.1223490 PMC4420145

[B28] Ives A. Ronet C. Prevel F. Ruzzante G. Fuertes-Marraco S. Schutz F. (2011). *Leishmania* RNA virus controls the severity of mucocutaneous leishmaniasis. *Science* 331 775–778. 10.1126/science.1199326 21311023 PMC3253482

[B29] Jenkins T. P. Brindley P. J. Gasser R. B. Cantacessi C. (2019). Helminth microbiomes: a hidden treasure trove? *Trends Parasitol.* 35 13–22. 10.1016/j.pt.2018.10.007 30503365

[B30] Kapheim K. M. Rao V. D. Yeoman C. J. Wilson B. A. White B. A. Goldenfeld N. (2015). Caste-specific differences in hindgut microbial communities of honey bees (*Apis mellifera*). *PLoS One* 10:e0123911 10.1371/journal.pbio.100123911 PMC4398325 25874551

[B31] Katoh K. Misawa K. Kuma K.-I. Miyata T. (2002). MAFFT: a novel method for rapid multiple sequence alignment based on fast Fourier transform. *Nucl. Acids Res.* 30 3059–3066. 10.1093/nar/gkf436 12136088 PMC135756

[B32] Knights D. Kuczynski J. Charlson E. S. Zaneveld J. Mozer M. C. Collman R. G. (2011). Bayesian community-wide culture-independent microbial source tracking. *Nat. Methods* 8 761–763. 10.1038/nmeth.1650 21765408 PMC3791591

[B33] Koch H. Schmid-Hempel P. (2012). Gut microbiota instead of host genotype drive the specificity in the interaction of a natural host-parasite system. *Ecol. Lett.* 15 1095–1103. 10.1111/j.1461-0248.2012.01831.x 22765311

[B34] Kolodny O. Weinberg M. Reshef L. Harten L. Hefetz A. Gophna U. (2019). Coordinated change at the colony level in fruit bat fur microbiomes through time. *Nat. Ecol. Evol.* 3 116–124. 10.1038/s41559-018-0731-z 30532043

[B35] Lagrue C. Poulin R. (2007). Life cycle abbreviation in the trematode *Coitocaecum parvum*: can parasites adjust to variable conditions? *J. Evol. Biol.* 20 1189–1195. 10.1111/j.1420-9101.2006.01277.x 17465928

[B36] Lahti L. Shetty S. (2012–2019). *Microbiome R Package.* Available online at: http://microbiome.github.io 10.1111/j.1420-9101.2006.01277.x (accessed September 21, 2019)

[B37] Leclair M. Pons I. Mahéo F. Morlière S. Simon J. C. Outreman Y. (2016). Diversity in symbiont consortia in the pea aphid complex is associated with large phenotypic variation in the insect host. *Evol. Ecol.* 30 925–941.

[B38] Leff J. W. (2017). *Mctoolsr: Microbial Community Data Analysis Tools. R Package Version 0.1.1.2.* Available online at: https://github.com/leffj/mctoolsr (accessed November 13, 2019).

[B39] Lemos L. N. Fulthorpe R. R. Triplett E. W. Roesch L. F. W. (2011). Rethinking microbial diversity analysis in the high throughput sequencing era. *J. Microbiol. Methods* 86 42–51. 10.1016/j.mimet.2011.03.014 21457733

[B40] Love M. I. Huber W. Anders S. (2014). Moderated estimation of fold change and dispersion for RNA-seq data with DESeq2. *Genome Biol.* 15:550. 10.1186/s13059-014-0550-8 25516281 PMC4302049

[B41] Lozupone C. Knight R. (2005). UniFrac: a new phylogenetic method for comparing microbial communities. *Appl. Environ. Microbiol.* 71 8228–8235. 10.1128/AEM.71.12.8228-8235.2005 16332807 PMC1317376

[B42] Lozupone C. A. Hamady M. Kelley S. T. Knight R. (2007). Quantitative and qualitative β diversity measures lead to different insights into factors that structure microbial communities. *Appl. Environ. Microbiol.* 73 1576–1585. 10.1128/AEM.01996-06 17220268 PMC1828774

[B43] Lynch J. B. Hsiao E. Y. (2019). Microbiomes as sources of emergent host phenotypes. *Science* 365 1405–1409. 10.1126/science.aay0240 31604267

[B44] Marotz C. Amir A. Humphrey G. Gaffney J. Gogul G. Knight R. (2017). DNA extraction for streamlined metagenomics of diverse environmental samples. *Biotechniques* 62 290–293. 10.2144/000114559 28625159

[B45] Martin M. (2011). Cutadapt removes adapter sequences from high-throughput sequencing reads. *EMBnet. J.* 17 10–12. 10.14806/ej.17.1.200

[B46] McFall-Ngai M. Hadfield M. G. Bosch T. C. G. Carey H. V. Domazet-Loso T. Douglas A. E. (2013). Animals in a bacterial world, a new imperative for the life sciences. *Proc. Nat. Acad. Sci. U.S.A.* 110 3229–3236. 10.1073/pnas.1218525110 23391737 PMC3587249

[B47] McMurdie P. J. Holmes S. (2013). phyloseq: an R package for reproducible interactive analysis and graphics of microbiome census data. *PLoS One* 8:e61217 10.1371/journal.pbio.10061217 PMC3632530 23630581

[B48] Oksanen J. Blanchet F. G. Friendly M. Kindt R. Legendre P. McGlinn D. (2019). *Vegan: Community Ecology Package. R Package Version 2.5-6.* Available online at: https://CRAN.R-project.org/package=vegan (accessed September 21, 2019).

[B49] Parada A. E. Needham D. M. Fuhrman J. A. (2016). Every base matters: assessing small subunit rRNA primers for marine microbiomes with mock communities, time series and global field samples. *Environ. Microbiol.* 18 1403–1414. 10.1111/1462-2920.13023 26271760

[B50] Pedregosa F. Varoquaux G. Gramfort A. Michel V. Thirion B. Grisel O. (2011). Scikit-learn: machine learning in Python. *J. Mach. Learn. Res.* 12 2825–2830.

[B51] Ponnusamy L. Willcox A. C. Roe R. M. Davidson S. A. Linsuwanon P. Schuster A. L. (2018). Bacterial microbiome of the chigger mite *Leptotrombidium imphalum* varies by life stage and infection with the scrub typhus pathogen *Orientia tsutsugamushi*. *PLoS One* 13:e0208327 10.1371/journal.pbio.100208327 PMC6283546 30521561

[B52] Poulin R. (2003). Information about transmission opportunities triggers a life history switch in a parasite. *Evolution* 57 2899–2903. 10.1111/j.0014-3820.2003.tb01530.x 14761067

[B53] Price M. N. Dehal P. S. Arkin A. P. (2010). FastTree 2 – approximately maximum-likelihood trees for large alignments. *PLoS One* 5:e9490. 10.1371/journal.pone.0009490 20224823 PMC2835736

[B54] R Core Team (2018). *R: A Language And Environment For Statistical Computing.* Vienna: R Foundation for Statistical Computing.

[B55] Roughgarden J. Gilbert S. F. Rosenberg E. Zilber-Rosenberg I. Lloyd E. A. (2018). Holobionts as units of selection and a model of their population dynamics and evolution. *Biol. Theory* 13 44–65.

[B56] Sinnathamby G. Henderson G. Umair S. Janssen P. Bland R. Simpson H. (2018). The bacterial community associated with the sheep gastrointestinal nematode parasite *Haemonchus contortus*. *PLoS One* 13:e0192164 10.1371/journal.pbio.100192164 PMC5805237 29420571

[B57] Takacs-Vesbach C. King K. Van Horn D. Larkin K. Neiman M. (2016). Distinct bacterial microbiomes in sexual and asexual *Potamopyrgus antipodarum*, a New Zealand freshwater snail. *PLoS One* 11:e0161050 10.1371/journal.pbio.10161050 PMC5001651 27563725

[B58] van den Bogert B. Meijerink M. Zoetendal E. G. Wells J. M. Kleerebezem M. (2014). Immunomodulatory properties of *Streptococcus* and *Veillonella* isolates from the human small intestine microbiota. *PLoS One* 9:e114277 10.1371/journal.pbio.100114277 PMC4257559 25479553

[B59] Van Vliet S. Doebeli M. (2019). The role of multilevel selection in host microbiome evolution. *Proc. Nat. Acad. Sci. U.S.A.* 116 20591–20597. 10.1073/pnas.1909790116 31548380 PMC6789794

[B60] Vaughan J. A. Tkach V. V. Greiman S. E. (2012). Neorickettsial endosymbionts of the Digenea: diversity, transmission and distribution. *Adv. Parasitol.* 79 253–297. 10.1016/B978-0-12-398457-9.00003-2 22726644

[B61] Vijayan N. Lema K. A. Nedved B. T. Hadfield M. G. (2019). Microbiomes of the polychaete *Hydroides elegans* (Polychaeta: Serpulidae) across its life-history stages. *Mar. Biol.* 166:19.

[B62] Vitetta L. Llewellyn H. Oldfield D. (2019). Gut dysbiosis and the intestinal microbiome: *Streptococcus thermophilus* a key probiotic for reducing uremia. *Microorganisms* 7:228. 10.3390/microorganisms7080228 31370220 PMC6723445

[B63] Wickham H. (2016). *Ggplot2: Elegant Graphics for Data Analysis.* New York: Springer-Verlag.

[B64] Wilkinson D. A. Duron O. Cordonin C. Gomard Y. Ramasindrazana B. Mavingui P. (2016). The bacteriome of bat flies (Nycteribiidae) from the Malagasy region: a community shaped by host ecology, bacterial transmission mode, and host-vector specificity. *Appl. Environ. Microbiol.* 82 1778–1788. 10.1128/AEM.03505-15 26746715 PMC4784053

[B65] Zilber-Rosenberg I. Rosenberg E. (2008). Role of microorganims in the evolution of animals and plants: the hologenome theory of evolution. *FEMS Microbiol. Rev.* 32 723–735. 10.1111/j.1574-6976.2008.00123.x 18549407

