# Supplementary material for: Persistence of a Core Microbiome Through the Ontogeny of a Multi-Host Parasite
Source: Front Microbiol. 2020 May 19;11:954. doi: 10.3389/fmicb.2020.00954 (PMC7248275; doi:10.3389/fmicb.2020.00954)
Supplement: Supplementary file 1 [file Data_Sheet_1.pdf]

Supplementary Material

Persistence of a core microbiome through the ontogeny of a multi-host parasite

Fátima Jorge, Nolwenn M. Dheilly and Robert Poulin

Extended Methods

Sample collection, processing and metadata

Naturally infected *C. parvum* hosts spanning the parasite ontogenetic development were searched among snails (*Potamopyrgus antipodarum*), amphipods (*Paracalliope fluviatilis*), and fish (*Gobiomorphus cotidianus*) collected from Lake Waihola, South Island, New Zealand during the 2019 austral summer. Samples were collected at two time points (3 days apart) under an approved Animal Use Protocol from the University of Otago (AUP-18-233). Immediately prior to animal collection, two types of environmental samples were collected, i.e. water (two samples) and lake sediment (two samples collected from where snails were collected), by immersing a sterile cotton swab and swirling it for a few seconds in lake water and substrate surface, respectively, breaking the tip and saving it in a PowerBead Pro Tube (QIAGEN), changing sterile gloves between each sample collection. Two controls for the swabs themselves were also taken by opening the swab and exposing it to natural air prior to saving it in a PowerBead Pro Tube. Environmental and control samples were snap frozen and kept in a -80ºC freezer. Waihola lake water was also collected into sterile containers for maintenance of specimens in the laboratory until processing.

In the laboratory, snails were placed in individual sterile wells with lake water, and incubated for two days at 25ºC under light to identify *C. parvum*-infected individuals through cercarial shedding. Amphipods were individually placed in sterile wells containing water and screened under the microscope for signs of infection (Lagrue & Poulin, 2007). Fish were kept alive in aerated lake water until further processing. Within each group, host and parasite specimens were a priori randomly selected using the function *sample* from the R package (R Core Team, 2018).

All dissections were conducted in a sterile laminar flow cabinet, and between each sample tools were cleaned with bleach, and sterilised with ethanol and burning flame. Prior to dissections, two samples were taken with sterile swabs of the water in which each host species were kept, to serve as controls for contamination within the laboratory environment. Snails were brushed with sterile interdental brush in 99% EtOH, and rinsed thoroughly in heat-sterilised PBS prior to dissections. From the eight infected snails, we successfully isolated sporocysts (two per snail from eight snails, n= 16), cercariae (three per snail from three of the eight snails, n = 9) and snail tissue

(adjacent to parasite tissue but free of it from five snails; n = 5). Amphipods were rinsed thoroughly in a series of 70%, and 99% EtOH, and then PBS. Metacercariae (1-3 per amphipod, n = 12) and amphipod tissue (whole body after parasite removal; n = 6) were collected. Fish were euthanised with an overdose of MS-222, and placed individually in sterile petri dishes. Before dissection, fish were brushed with Betadine (Sanofi) to prevent contamination of the body cavity with skin microbes. Their intestinal tract was aseptically removed from the abdominal cavity, and opened to find adult parasites. Adult worms (1-3 per fish, n = 10) and fish tissue (clean of parasites and contents, n = 5) were collected. All tissue samples, both parasite and host, were cleaned from surface microbiota by pipetting up and down in PBS in sterile wells. Samples of the surface micriobiota for each sample type was collected by pipetting 75ul of the resulting 'washing' (two samples per host type and parasite life stage). For each host group a sample of the PBS solution was taken at the end of the procedures to account for any possible contamination of the solution. Samples were snap frozen and kept in a -80ºC freezer until DNA isolation. Metadata on sample type (e.g. environmental, host type, parasite, controls), life stage (e.g. sporocyst, metacercaria), and host ID are given in Table S1.

Library preparation and Microbiome sequencing

DNA was extracted using the DNeasy PowerSoil Pro Kit (QIAGEN), following the manufacturer's protocol, with modifications recommended for cells difficult to lyse by the EMP DNA Extraction Protocol (Marotz et al., 2017). Together with the isolated biological samples, two ZymoBIOMICS microbial community standards samples (MCS: 75 µl and 37.5 µl) containing microbes of varying size and cell wall recalcitrance (eight bacteria and two yeasts), and one reagent-only sample were also extracted to assess the performance and contamination of our workflow, respectively. Order of extraction was randomised as described above, to the exception of the reagent-only sample which was last. All samples were eluted in 100 µl of solution C6 and stored at -20ºC until needed.

DNA libraries for each sample were prepared following EMP 16S Illumina Amplicon Protocol to amplify prokaryotes using paired-end community sequencing. The V4 hypervariable region of the prokaryotic bacterial 16S SSU rRNA gene was PCR-amplified and multiplexed using the universal bacterial primers 515F (Parada) - 806R (Apprill) (Apprill et al., 2015; Parada et al., 2016). Together with the biological samples of interest, one additional control sample of 0.2ng of the ZymoBIOMICS microbial community DNA standards (MCS DNA) and a reagent-only sample were also included. Again, sample order on the PCR plate was randomized, with the exception of the reagent-only sample PCR sample which was last. Samples were amplified in triplicate in a 20 µl mix composed of 5.6 µl of ultrapure water, 10 µl of MyFi^{TM} mix (Bioline), 8 µM of each primer and 2 µl of DNA template. The PCR conditions consisted of an initial denaturation step of 3 min at

95°C and 35 cycles, each consisting in one cycle of 45 s at 95°C, 60 s at 50°C, and 90 s at 72°C, followed by a final extension cycle of 10 min at 72ºC. Triplicate libraries of each sample were pooled and run on a 2% agarose gel. We then used a quantitative binding approach to clean and normalise each amplicon with SequalPrep Kit (Invitrogen) following the manufacturer's protocol. This protocol requires that DNA is present in excess (≥250 ng) for accurate normalization; given that several samples were below this requirement, we quantified DNA concentration with QuBit with 1X dsDNA HS Assay Kit (Invitrogen). Each amplicon library was then manually diluted to the lowest measured concentration of biological samples, and equal volumes of amplicons were combined in a single tube to construct the final libraries pool. Due to the low concentration expected (~0.2 ng/µl), 283.66 µl of the pool was concentrated to 107 µl using a Concentrator Plus (Eppendorf) at 45ºC for 30 min. The DNA concentration of this pool of libraries was quantified with QuBit (as above), and the average molecule length was determined using the Agilent 2100 bioanalyzer instrument (Agilent DNA 1000 Reagents). Combined barcoded libraries were sequenced on an Illumina MiSeq platform using the V2 reagent cartridge (250 bp, paired-end) through Otago Genomics & Bioinformatics Facility (New Zealand).

Sequence processing

Data were received as demultiplexed paired-end raw sequences, and were processed and analysed using the Quantitative Insights Into Microbial Ecology (QIIME) 2 software package (Bolyen et al., 2019). Adapters and primers were removed from raw sequences using the plugin *cutadapt* (with 0 error-rate and minimum length of 240 bp) (Martin, 2011), and quality filtered using *dada2* plugin (Callahan et al., 2016) after inspection of quality profile plots of forward and reverse reads. Based on quality profiles, the first thirteen bases and last ten nucleotides of the forward and reverse were trimmed to avoid errors typically associated with these regions and improve the *dada 2* algorithm's sensitivity. The resulting amplicon sequence variants (ASVs) were then filtered to remove non-bacterial contaminant sequences (i.e. host sequences). Exclusion was determined by alignment using threshold values of 0.8 identity and 0.8 query alignment against the Greengenes 16S rRNA reference sequences (99 OTUS, 13_8 release) using the *quality-control* plugin. ASVs excluded were checked to determine their identity in BLAST. ASVs corresponding to mitochondria or chloroplast, or without a phylum assignment were further excluded from the data. Contaminants were removed by filtering ASVs found in blanks (e.g. PCR blank, swab control, PBS controls and extraction blank) using the *feature-table* plugin. While this methodology does not remove contaminants which could possibly originate from cross-contamination during sample processing, data filtered with *decontam* R package (Davis et al., 2017) contained more contaminants as assessed in the MCS quality check analyses (data not shown). We further explored potential sources of

contamination during the incubation period of snail, amphipod and fish by excluding all ASVs present exclusively in laboratory water controls as identified by comparing with ASVs from natural environmental samples (using the function 'anti-join' from the *dplyr* R package v. 0.8.3 (Wickham et al., 2019), and using the *feature-table* plugin from QIIME2). Finally, the obtained feature table was filtered to remove samples with low sequencing depth (i.e. frequency lower than 1,000, and/or with less than 8 features). A 'reduced dataset' which did not include ASVs not shared by at least two samples (*feature-table* plugin) was also created. For analyses regarding the diversity of parasite-associated microbial communities, these two datasets were further filtered to include only those samples extracted from parasite tissues. For each dataset different taxonomic levels were assigned to the ASVs using the plugin *feature-classifier* (Bokulich et al., 2018) against the Greengenes 16S rRNA reference database (13_8 release) pre-trained on the 515F/806R region (Pedregosa et al., 2011). Taxonomic barplots were created with the plugin *taxa barplot ()*.

Sequenced data quality was evaluated based on the observed composition and sequence quality of the ZymoBIOMICS microbial community standards (MCS and MCS DNA), against the expected data of these mock communities, using *q2-quality-control* plugin. This analysis allowed us to assess how well our methods and pipeline estimate the microbial community present in the samples. The analyses were performed on data still with contaminants found in blanks and without them (see above for methods for exclusion of contaminants). First, we estimated if there were any mismatches between the observed sequences and the set of MSC reference sequences as a measure of sequencing errors. Then, we assessed the level of community accuracy by comparing the observed and expected sample composition. These analyses also allowed us to determine taxonomic classification accuracy, i.e the lowest taxonomic level as determined by the taxonomic classifier that was correctly attributed or below which there were under-classifications.

Diversity analyses.

Diversity analyses were performed primarily using QIIME 2, and the R packages vegan v. 2.5-6 (Oksanen et al., 2019) and phyloseq v. 1.22.3 (McMurdie & Holmes, 2013) with default function settings unless otherwise noted. Prior to analyses, ASVs were aligned using the mafft program (Katoh et al., 2002) and used to construct a phylogenetic tree using the fasttree2 program (Price et al., 2010) using the *phylogeny* plugin. For analysis, the filtered ASVs and taxonomy tables, and the rooted tree were imported into R with the qiime2R package v. 0.99.12 (Bisanz, 2018) and together with the metadata combined into a phyloseq object. Given that one of the sources of potential 'noise' in metabarcoding analysis is the fine-scale data (here ASVs), analyses were also performed at the higher taxonomic ranks Phylum and Family using the agglomeration *phyloseq* function tax_glom. Phyloseq objects were evenly subsampled using *rarefy_even_depth()*.

We started by investigating the presence of a 'core' microbiome common to all parasite life stages, and specific to each life stage. First, Venn diagrams were created at the family level with an online tool (http://bioinformatics.psb.ugent.be/webtools/Venn/). We tested for the presence of a 'core' microbiome as defined by any taxon with a prevalence higher than 0.95, 0.75 or 0.50 with the *microbiome* package v. 0.99.87 (Lahti & Shetty, 2012-2019) with the *core* function (detection = 0). Core heatmaps were created with the function *plot_core()* using absolute counts. To infer which families had a higher relative abundance among life stages, we created heatmaps using *plot_ts_heatmap()* of the mctoolsr package v. 0.1.1.2 (Leff, 2017). A tree plot was created over the full tree estimated from the alignment of parasite ASVs using *plo_tree()* to visualise how microbiota components of the different life stages relate to each other, and how the life stages relate to each other.

The diversity within each parasite life stage (alpha diversity) was calculated using the following metrics: Faith's phylogenetic diversity, evenness and Shannon using the QIIME 2 *alpha-group-significance* plugin. The Kruskal–Wallis test was used to calculate pairwise comparisons between alpha diversity estimates among life stages.

To test whether life stages differ in community composition (beta diversity), we used phylogenetic-based indices which are useful even with low sequence coverage (Lemos et al., 2011), but also given that phylogenetic information is relevant to the questions in our study. Specifically, the qualitative unweighted Unifrac (Lozupone & Knight, 2005) and quantitative weighted UniFrac (Lozupone et al., 2007) distance metrics were calculated with *distance()*. First, to explore the structure of microbial communities, principal coordinates plots (PCoA) were created with *plot_ordination()* adding hulls as defined with *find_hull()* from the erictools package (https://rdrr.io/github/elittmann/erictools/man/find_hull.html). Statistically significant differences among life stages were determined with permutational ANOVA performed with *adonis* and with multilevel pairwise comparisons with *pairwise.adonis()* with Benjamini and Hochberg's (1995) ("BH-FDR") correction for multiple testing with 9999 permutations. Permutational ANOVA assumes that there is sufficient homogeneity of dispersion within sample types. This was evaluated with *betadisper()*. We further explored if there were differential abundances of bacterial phylotypes between consecutive life stages with DESeq2 v. 1.18.1 (Love et al., 2014). For this the non-rarefied phyloseq object was used as input into DESeq2 for differential abundance analysis with *phyloseq_to_deseq2()* and using geoMeans to estimate size factors. DESeq() was called with default parameters, and results were contrasted by life stage, and an adjusted p-value cut-off of 0.05 was used for differences in relative abundances to be considered statistically significant.

Sources of parasite microbiome

We tested whether the parasite's microbial composition differed from that of its different hosts and environment using the same diversity analyses as described above. Using Venn diagrams, we determined if there were any taxa unique to the parasite bacterial community, irrespective of their abundances and prevalence.

To determine the likely main sources of each life stage microbiome, we used the Bayesian approach SourceTracker developed for R (Knights et al., 2011). For each parasite life stage (classified as 'sink'), we used SourceTracker to estimate the proportion of bacteria originating from potential 'sources': environmental samples (water and sediment, and laboratory environment), the host, the prior parasite life stage, or unknown sources (representing one or more sources absent from the training data) using the ASV data of the reduced dataset with a rarefaction of 1,000. Samples were classified as sources (potential contributors to a given microbial community) or sinks (the community being investigated), and a total of four analyses were conducted (one per life stage). Alpha values were first tuned using cross-validation with *tune.st()*, and SourceTracker object was then trained using samples identified as source with *sourcetracker()*. We then predicted for each sink and their respective sources training data, the proportional contribution of sources with *predict()*. Bar plots were created over the mean and standard deviation of the resulting proportion estimates of contributing sources for each parasite life stage, and also for the respective train data with ggplot2 v. 3.2.1 (Wickham, 2016).

Supplementary Results

Quality control: Is there any bias in DNA extraction and/or sequence quality?

The protocol blank samples (n = 7) contained 102 ASVs (58 of which unique to the blanks) which were all removed from the sample dataset. Additionally, 813 ASVs were found in the water where samples were held in the laboratory at the time of isolation, but not present in the natural environment, and as a consequence were also excluded from the dataset.

Quality control analyses based on the observed and expected MSC (performed on data still with blank features), did not detect bias in extraction and amplification of the different bacteria composing the MSC, since all eight bacteria (three Gram-negative and five Gram-positive) were successfully amplified. Sequence quality analysis did not detect any errors in sequencing, since all expected MSC were observed with zero sequence mismatches (Fig. S1A, Table S8). Analysis performed on data which still included features found in blanks, revealed three additional features that were classified as contaminants. However, after exclusion of contaminants found in blanks, four component taxa of MSC community composition were missing, while one taxon classified as a false positive (possibly a contaminant) was still present in the data. Taxonomic assessment based on Greengenes 16S rRNA reference database revealed bias in classification below the family level with 7 of the nine features being underclassified (Fig S1B). Given this result, only family level classification is given even when referring to ASVs.

(A) (B)

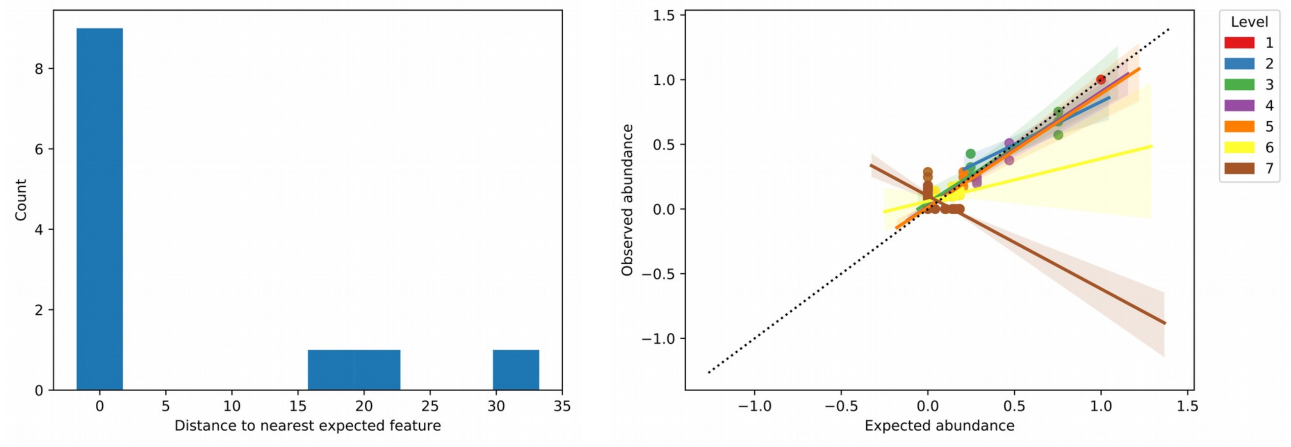

Figure S1. (A) Mismatches between false positives and expected ASVs from the raw data (post dada 2 processing, but prior to removal of contaminants). The eight expected taxa from the ZymoBIOMICS microbial community standards were all found (two strands of *Salmonella enterica*, 16S_1 and 16S_6 were retrieved) with 100% percentage of identity and zero mismatches between observed and expected sequences. The three false positives were observed. (B) Linear regression between observed and expected abundances based on classification with Greengenes 16S rRNA reference sequences (99 OTUS, 13_8 release).

Does the microbial community of the parasite differ from that of the environment and hosts?

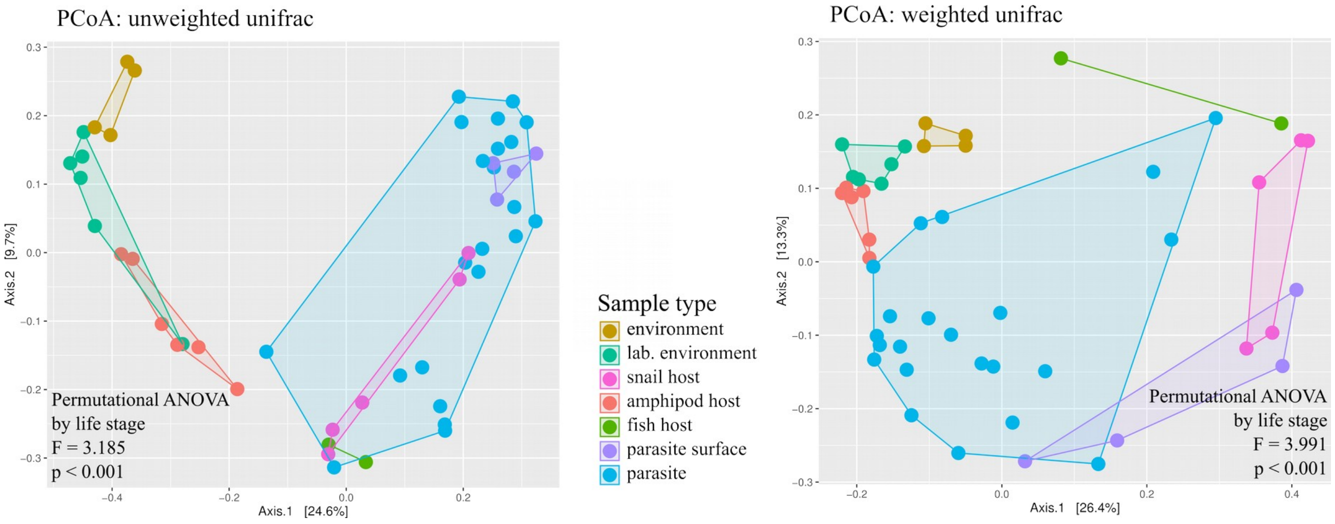

Figure S2. Principal coordinates analyses ordinations based on unweighted and weighted unifrac over the reduced dataset, with hulls delimiting each life stage group of samples; results of permutational ANOVA test also shown.

Parasite 'core' microbiome — Reduced dataset

For the reduced dataset, only one family had a prevalence higher than 0.5 (Comamonadaceae) across all microbial communities in parasite samples.

Life stage-specific 'core' analysis revealed higher prevalence values at a finer scale (i.e. ASVs), between 0.75 and 0.5 for all life stages in the reduced dataset with the exception of cercariae [reduced dataset: three for sporocysts (Bradyrhizobiaceae, Methylobacteriaceae and Comamonadaceae), none for metacercariae, and two for adult worms (Comamonadaceae and Bacillaceae)].

Comparing microbial abundance between life stages

Pairwise comparisons between consecutive life stages identified from 195 ASVs with nonzero read count that between adult and metacercaria, one taxon was significantly less abundant in metacercaria stage (Ruminococcaceae; for the reduced dataset there were four out of 49 with nonzero read count: Ruminococcaceae sp., Chitinophagaceae, Neisseriaceae and Corynebacteriaceae). Only in estimates based on the reduced dataset did we find differential abundances between adult and sporocyst stage, where the adult had one taxa out of 49 with higher abundance (Ruminococcaceae). Estimation of differential abundance at family level revealed that

from 80 families with nonzero read count, cercariae have significantly higher abundance of Cryomorphaceae than metacercariae, and again metacercariae had significantly lower abundance of Ruminococcaceae and Fusobacteriaceae (for the reduced dataset only Ruminococcaceae) than in the adult stage.

Comparing microbial diversity between inside versus outside of parasites

There were no significant differences between the diversity of bacterial communities of each parasite life stage and those of their respective surface microbiota, with the exception of metacercariae in which the surface microbiota had higher evenness (H =4.418, p = 0.036, but BH-FDR = 0.177). Similarly, using principal coordinate analysis on unweighted and weighted Unifrac distances, no difference in microbial communities was found between the four life stages and the microbiota living on their outer surfaces (p > 0.05).

Which is the main source of each parasite life stage?

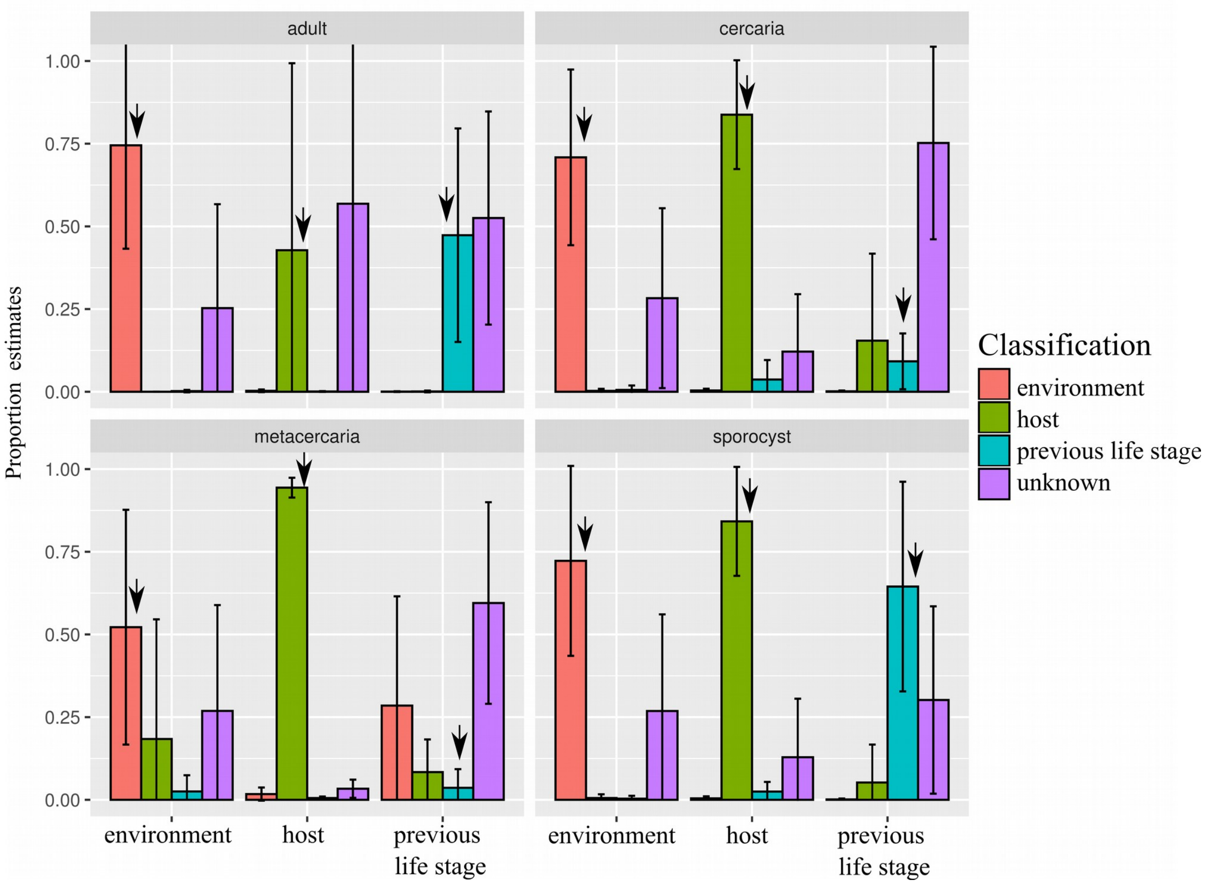

Figure S3. Results from SourceTracker source samples training. For each source in each analysis, the proportion of correct assignment to the known identity or other sources is given. Arrows indicate the correct classification for each source during training; in most cases, the correct source was the most frequently assigned.

Supplementary References

Apprill, A., McNally, S., Parsons, R., & Weber, L. (2015). Minor revision to V4 region SSU rRNA 806R gene primer greatly increases detection of SAR11 bacterioplankton. *Aquatic Microbial Ecology,* 75, 129-137.

Benjamini, Y., & Hochberg, Y. (1995). Controlling the false discovery rate: a practical and powerful approach to multiple testing. *Journal of the Royal Statistical Society B,* 57, 289-300.

Bisanz, J. E. (2018). qiime2R: Importing QIIME2 artifacts and associated data into R sessions. https://github.com/jbisanz/qiime2R

Bokulich, N. A., Kaehler, B. D., Rideout, J. R., Dillon, M., Bolyen, E., Knight, R., … Gregory, C. J. (2018). Optimizing taxonomic classification of marker-gene amplicon sequences with QIIME 2's q2-feature-classifier plugin. *Microbiome,* 6, 90. https://doi.org/10.1186/s40168-018-0470-z

Bolyen, E., Rideout, J. R., Dillon, M. R., Bokulich, N. A., Abnet, C. C., Al-Ghalith, G. A., … Caporaso, J. G. (2019). Reproducible, interactive, scalable and extensible microbiome data science using QIIME 2. *Nature Biotechnology,* 37, 852–857.

Callahan, B. J., McMurdie, P. J., Rosen, M. J., Han, A. W., Johnson, A. J. A., & Holmes, S. P. (2016). DADA2: high-resolution sample inference from Illumina amplicon data. *Nature Methods,* 13, 581-583.

Davis, N. M., Proctor, D., Holmes, S. P., Relman, D. A., & Callahan, B. J. (2017). Simple statistical identification and removal of contaminant sequences in marker-gene and metagenomics data. *Microbiome,* 6, 226. https://doi.org/10.1186/s40168-018-0605-2

Katoh, K., Misawa, K., Kuma, K.-I., & Miyata, T. (2002). MAFFT: a novel method for rapid multiple sequence alignment based on fast Fourier transform. *Nucleic Acids Research,* 30, 3059-3066. https://doi.org/10.1093/nar/gkf436

Knights, D., Kuczynski, J., Charlson, E. S., Zaneveld, J., Mozer, M. C., Collman, R. G., … Kelley, S. T. (2011). Bayesian community-wide culture-independent microbial source tracking. *Nature Methods,* 8, 761-763. doi:10.1038/nmeth.1650

Lagrue, C., & Poulin, R. (2007). Life cycle abbreviation in the trematode *Coitocaecum parvum*: can parasites adjust to variable conditions? *Journal of Evolutionary Biology,* 20, 1189-1195.

Lahti, L., & Shetty, S. (2012-2019). microbiome R package. http://microbiome.github.io

Leff, J. W. (2017). mctoolsr: Microbial Community Data Analysis Tools. R package version 0.1.1.2. https://github.com/leffj/mctoolsr

Lemos, L. N., Fulthorpe, R. R., Triplett, E. W., & Roesch, L. F. W. (2011). Rethinking microbial diversity analysis in the high throughput sequencing era. *Journal of Microbiological Methods,* 86, 42-51.

Love, M. I., Huber, W., & Anders, S. (2014). Moderated estimation of fold change and dispersion

for RNA-seq data with DESeq2. *Genome Biology,* 15, 550.

Lozupone, C., & Knight, R. (2005). UniFrac: a new phylogenetic method for comparing microbial communities. *Applied and Environmental Microbiology,* 71, 8228-8235. doi: 10.1128/AEM.71.12.8228-8235.2005

Lozupone, C. A., Hamady, M., Kelley, S. T., & Knight, R. (2007). Quantitative and qualitative β diversity measures lead to different insights into factors that structure microbial communities. *Applied and Environmental Microbiology,* 73, 1576-1585. doi: 10.1128/AEM.01996-06

Marotz, C., Amir, A., Humphrey, G., Gaffney, J., Gogul, G., & Knight, R. (2017). DNA extraction for streamlined metagenomics of diverse environmental samples. *BioTechniques,* 62, 290-293.

Martin, M. (2011). Cutadapt removes adapter sequences from high-throughput sequencing reads. *EMBnet.journal,* 17, 10-12. http://dx.doi.org/10.14806/ej.17.1.200

McMurdie, P. J., & Holmes, S. (2013). phyloseq: An R package for reproducible interactive analysis and graphics of microbiome census data. *PLoS ONE,* 8, e61217.

Oksanen, J., Blanchet, F. G., Friendly, M., Kindt, R., Legendre, P., McGlinn, D., … Wagner, H. (2019). vegan: Community Ecology Package. R package version 2.5-6. https://CRAN.R-project.org/package=vegan

Parada, A. E., Needham, D. M., & Fuhrman, J. A. (2016). Every base matters: assessing small subunit rRNA primers for marine microbiomes with mock communities, time series and global field samples. *Environmental Microbiology,* 18, 1403-1414.

Pedregosa, F., Varoquaux, G., Gramfort, A., Michel, V., Thirion, B., Grisel, O., … Duchesnay, E. (2011). Scikit-learn: machine learning in Python. *Journal of Machine Learning Research,* 12, 2825-2830.

Price, M. N., Dehal, P. S., & Arkin, A. P. (2010). FastTree 2 – approximately maximum-likelihood trees for large alignments. *PLoS ONE,* 5, e9490. https://doi.org/10.1371/journal.pone.0009490

R Core Team. (2018). R: A language and environment for statistical computing. R Foundation for Statistical Computing, Vienna, Austria. https://www.R-project.org/

Wickham, H. (2016). *ggplot2: Elegant graphics for data analysis*. New York: Springer-Verlag.

Wickham, H., François, R., Henry, L., & Müller, K. (2019). dplyr: A Grammar of Data Manipulation. R package version 0.8.3. https://CRAN.R-project.org/package=dplyr
